# Differences in Pulmonary Artery Stiffness Measured by CMR in Preterm-Born Young Adults With and Without Bronchopulmonary Dysplasia

**DOI:** 10.1161/CIRCIMAGING.124.017791

**Published:** 2025-03-13

**Authors:** Wouter J. van Genuchten, Jarno J. Steenhorst, Gabrielle M.J.W. van Tussenbroek, Nikki van der Velde, Lieke S. Kamphuis, Irwin K.M. Reiss, Daphne Merkus, Willem A. Helbing, Alexander Hirsch

**Affiliations:** 1 Division of Pediatric Cardiology, Department of Pediatrics, Cardiovascular Institute, Erasmus MC, Rotterdam, The Netherlands (W.J.v.G., J.J.S., G.M.J.W.v.T., W.A.H.).; 2 Department of Cardiology, Cardiovascular Institute, Thorax Center, Erasmus MC, Rotterdam, The Netherlands (J.J.S., N.v.d.V., D.M., A.H.).; 3 Department of Radiology and Nuclear Medicine (J.J.S., N.v.d.V., W.A.H., A.H.), Erasmus MC, Rotterdam, The Netherlands.; 4 Department of Pulmonology (L.S.K.), Erasmus MC, Rotterdam, The Netherlands.; 5 Division of Neonatology, Department of Neonatal and Pediatric Intensive Care (I.K.M.R.), Erasmus MC, Rotterdam, The Netherlands.; 6 Walter-Brendel Centre of Experimental Medicine, LMU Munich, University Hospital Munich, Germany (D.M.).

**Keywords:** bronchopulmonary dysplasia, magnetic resonance imaging, preterm infants, pulmonary artery, pulmonary arterial hypertension

## Abstract

**BACKGROUND:**

Very preterm-born infants are at risk for developing bronchopulmonary dysplasia (BPD), a chronic lung disease. Nowadays, the majority of these infants reach adulthood. Very preterm-born young adults are at risk for developing pulmonary arterial (PA) hypertension later in life. An early sign of PA hypertension is increased PA stiffness. This study aims to use cardiovascular magnetic resonance to compare PA stiffness using PA relative area change (RAC) and pulse wave velocity (PWV) to identify early signs for PA hypertension in young adults born very premature, with and without BPD.

**METHODS:**

Twenty preterm-born young adults with and 20 without BPD underwent cardiovascular magnetic resonance and were compared with 20 at-term-born young adults. RAC was calculated as the percentage change between the maximal and minimal areas of the PA. PWV was calculated using a method that simultaneously compares flow and area increase in the pulmonary artery during early systole.

**RESULTS:**

In 57 of 60 patients, PWV and RAC measurements could be performed. Preterm-born young adults with BPD showed increased PWV compared with preterm-born young adults without BPD (median [25th–75th percentile] 2.07 m/s [1.45–3.05] versus 1.61 m/s [1.18–1.85]; *P*=0.04) and at-term-born young adults (1.35 m/s [1.08–2.23]; *P*=0.04). RAC was decreased in both preterm-born young adults with (62% [56–82]; *P*<0.01) and without BPD (78% [67–93]; *P*<0.01), compared with at-term-born young adults (101% [87–122]).

**CONCLUSIONS:**

Preterm-born young adults with BPD show increased PA stiffness as measured by PWV compared with preterm-born young adults without BPD and at-term-born young adults; RAC was decreased in both preterm-born groups compared with at-term controls. This noninvasive method of measuring PA stiffness might be a valuable tool to identify individuals at risk for early signs of PA hypertension in this population.

**CLINICAL PERSPECTIVE**

Preterm-born infants are at risk for developing bronchopulmonary dysplasia, a chronic lung disease characterized by a developmental arrest of the alveoli and pulmonary microcirculation. Nowadays, the majority of very preterm-born infants reach adulthood. These young adults are at risk for developing early cardiovascular disease, including pulmonary arterial hypertension. Pulmonary arterial hypertension is a detrimental disease, often with a long diagnostic delay, where early recognition is crucial to ensure therapeutic options. There is a need for noninvasive screening methods to identify high-risk individuals to start early treatment and prevention of secondary hits such as diabetes, smoking, and chronic kidney disease to improve outcomes. One of the early hallmarks of pulmonary arterial hypertension is increased pulmonary arterial stiffness, which can be measured by measuring the pulse wave velocity in the pulmonary artery using cardiovascular magnetic resonance. Preterm-born young adults with bronchopulmonary dysplasia show increased pulmonary arterial stiffness as measured by pulse wave velocity compared with preterm-born young adults without bronchopulmonary dysplasia and at-term-born young adults. Relative area change was decreased in both preterm-born groups compared with at-term controls.

About 1% of adults are born prematurely before 30 weeks of gestational age, and this percentage is increasing.^1^ This population is at high risk for bronchopulmonary dysplasia (BPD), a chronic neonatal lung disease.^2^ BPD is characterized by a developmental arrest in alveolarization and vascularization of the lungs.^3^ The survival of very and extremely premature-born patients has improved significantly due to improved treatment options, such as the introduction of exogenous administration of surfactant, which became a widespread standard of care after 1993.^4^ These developments result in increasing adult populations born very prematurely. These preterm young adults are at increased risk for cardiopulmonary diseases later in life,^5^ including a 5× increased risk of the development of pulmonary arterial hypertension (PAH) in adolescence.^6^ Preterm young adults with BPD subjected to invasive measurements showed (subclinical) increased pulmonary arterial pressures and increased pulmonary arterial stiffness, indicating early pulmonary vascular disease.^5^ Treatment options for overt adult PAH remain limited. Therefore, early detection of pulmonary vascular disease is crucial. However, invasive catheterization is not feasible for serial follow-up; therefore, noninvasive measurement of pulmonary artery (PA) stiffness is important.^7^ Pulse wave velocity (PWV) is a measurement of vessel stiffness and is frequently measured in the aorta. However, PWV can also be assessed in the proximal PA. In patients with PAH, invasively measured PA pressures correlated strongly with PWV, as measured by cardiovascular magnetic resonance (CMR).^8^ Our study aims to investigate if preterm-born young adults with or without BPD have increased pulmonary vascular stiffness, measured by CMR, compared with each other and to individuals born at term.

## Methods

The data that support the findings of this study are available from the corresponding author upon reasonable request. This study is part of a larger study investigating preterm-born young adults with and without BPD.^9^ Sixty young adults were included: 20 preterm-born subjects with BPD (median gestational age, 27 weeks [25th–75th percentile, 26–28]), 20 preterm-born subjects without BPD (gestational age, 28 weeks [27–29]), and 20 born at term (gestational age, 39 weeks [38–40]; age- and sex-matched). All participants were born after 1993. Preterm was defined as <30 weeks of gestational age. BPD was defined as postnatal oxygen dependency ≥28 days. Preterm-born subjects with and without BPD were recruited using the database of the Neonatal Intensive Care Unit of Erasmus MC Sophia Children’s Hospital, Rotterdam, the Netherlands. Patients with known hemodynamically significant heart disease (except as a consequence of PAH), pulmonary disorders other than BPD, kidney disorders, or neurodevelopmental disabilities that would prevent cooperation during cardiopulmonary tests are excluded. The protocol was approved by the medical ethics board of the Erasmus MC (MEC2016-427).

**Figure 1.**
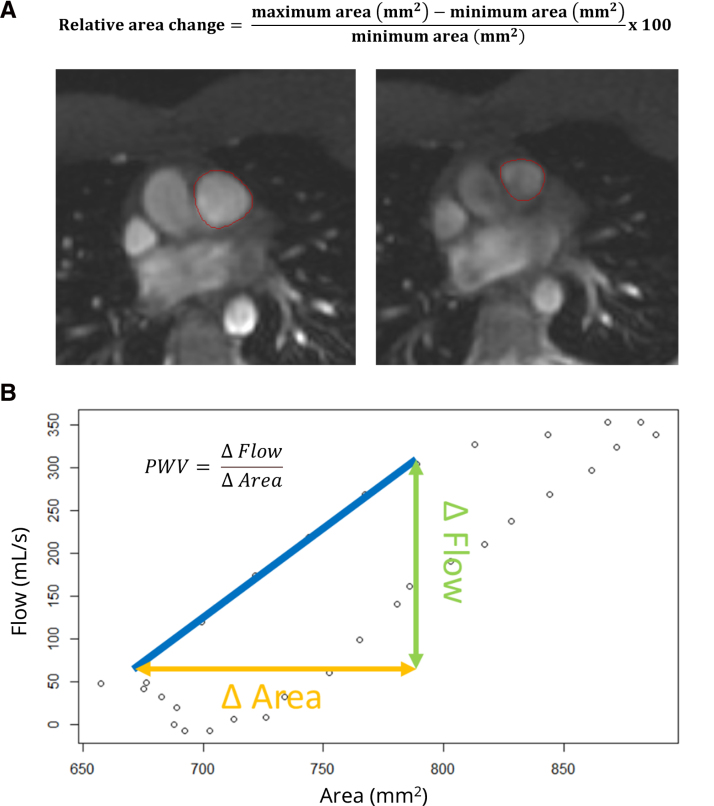
**Example of cardiovascular magnetic resonance-derived pulmonary artery stiffness measurements. A**, Relative area change measurement. **B**, Pulse wave velocity (PWV) measurement.

After informed consent, CMR was performed on a 1.5T clinical magnetic resonance imaging system (SIGNA Artist; GE Healthcare, WI). Retrospectively ECG-gated scans were performed and included a standard stack of short-axis balanced steady-state free precession cine imaging acquired during end-expiration breath-hold for ventricular volumes and function. Typical scan parameters were 1 slice per breath-hold, slice thickness of 6 mm, interslice gap of 4 mm, repetition time of 3.8 ms, echo time of 1.7 ms, flip angle of 65°, number of excitations 1, array spatial sensitivity encoding technique of 2, views per segment of 14, field of view of 360×288 mm, and acquired matrix of 200×280 with 30 reconstructed phases per cardiac cycle. Retrospectively, ECG-gated free-breathing phase contrast imaging of the PA (≈1 cm above the valve proximal to bifurcation) was performed with a field of view of 340–380 mm, slice thickness of 7 mm, matrix size of 192×128, flip angle of 20°, echo time of 3.5 ms, repetition time of 5.8 ms, array spatial sensitivity encoding technique of 1.5, number of excitations 2, VENC of 180 cm/s, views per segment of 2, temporal resolution of 23 ms, and 60 reconstructed phases per cardiac cycle. CMR analyses were done using commercially available postprocessing software (Qmass & Qflow software, version 8.1; Medis Medical Imaging, Leiden, the Netherlands). All images were anonymized and analyzed in a random order blinded to the subject group. To measure left and right ventricular volumes, ejection fraction, and mass, epi- and endocardial contours of the left and right ventricles were drawn in end-diastole and end-systole (papillary muscles and trabecula included in volume). PA stiffness was approximated by PWV and relative area change (RAC) measurements. PWV was measured with the method developed by the University of Colorado.^10^ This method utilizes the relation between increase in flow and increase in area: $PWV=\frac{\Delta \;Flow}{\Delta \;Area}$ in the first linear part of systole. A straight line was drawn from the start of systole to the point where the flow-area curve deviates from the linear area-flow relationship. This point was defined as the end of early systole. By including only the early systole, underestimation of PWV that could be caused by wave reflections is minimized^10^ (Figure 1A). To assess pulmonary area and flow volume, the PA was manually delineated in at least 1 cardiac phase. Automatic border detection was used for the other cardiac phases. These contours were reviewed and adapted manually when necessary for each cardiac phase using the magnitude and phase contrast images. Both area and flow were interpolated to a 1 ms resolution using the zoo package in R software (version 4.4.0).^11,12^ Afterward, the linear part of the increase during early systole was used to calculate PWV (Figure 1A). The RAC was calculated as $\;\frac{maximum\;area\;\left(mm^{2}\right)-\;minimum\;area\;\left(mm^{2}\right)}{minimum\;area\;\left(mm^{2}\right)}\;\;\;\times \;\;\;100$ of the PA using the contours drawn in the phase contrast images (Figure 1B). Furthermore, the ventricular curvature ratio was measured as described by Critser et al.^13^ Ventricular curvature ratio was measured on the cine images in the short-axis plane at the level of the left ventricular papillary muscles using ImageJ software (National Institutes of Health, Bethesda, MD). The ventricular curvature ratio was determined from the ratio of the curvature of the interventricular septum and left ventricular lateral wall. Finally, cardiopulmonary exercise testing was performed on an upright cycling ergometer as described earlier.^9^ Analyses were done using R software (version 4.4.0).^11^ Considering the small group size, all variables were assumed not normally distributed and described using the median with 25th to 75th percentiles. To compare all 3 groups, the Kruskal-Wallis test and χ^2^ test were used. When pairwise comparisons were made, the Mann-Whitney *U* test was used. Linear nonparametric correlation was calculated using the Spearman correlation.

## Results

**Figure 2.**
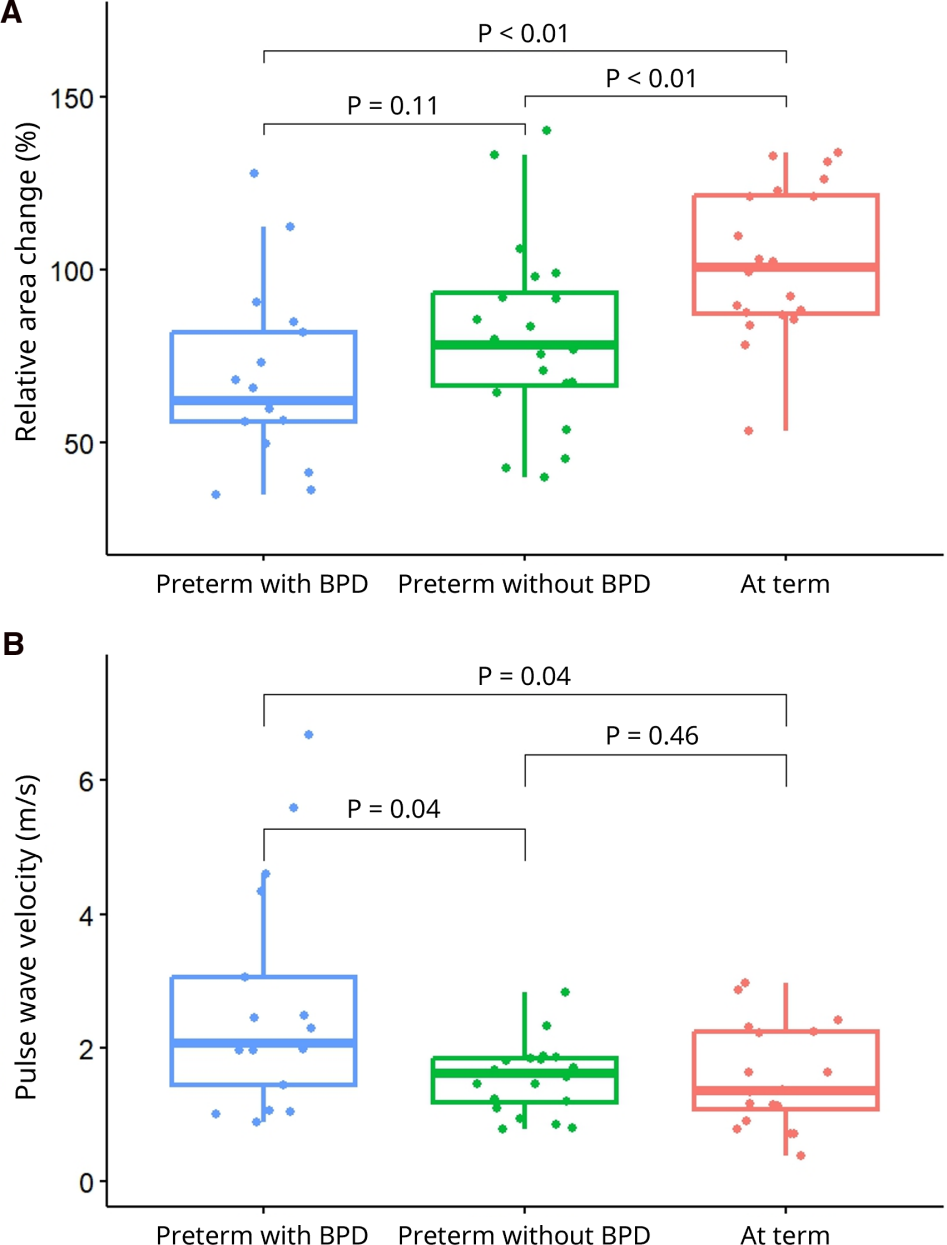
**Comparison of cardiovascular magnetic resonance-derived pulmonary artery stiffness measurements between preterm-born young adults with and without bronchopulmonary dysplasia (BPD) compared with controls born at term. A**, Relative area change. **B**, Pulse wave velocity.

PWV and RAC measurements of the PA could be performed from 57 of the 60 CMRs. In 3 preterm-born subjects with BPD, no accurate contours could be drawn due to inadequate signal quality. The baseline parameters for all groups are displayed in the Table. The median age of the 57 years included individuals was 22 (25th-75th percentile, 21–25) years and 31 (54%) were female. No differences were found for age, sex, body mass index (23 kg/m^2^ [21–24]), and body surface area (1.85 m^2^ [1.71–1.97]) between the 3 groups. Days of oxygen dependency were highest in preterm-born subjects with BPD, in between in preterm-born subjects without BPD, and lowest in subjects born at term. All these differences were statistically significant. There was no significant difference in gestational age between preterm-born young adults with and without BPD (*P*=0.29). Maximum workload was significantly different between preterm-born adults with BPD, without BPD, and at-term controls (*P*=0.01). Left and right ventricular end-diastolic volumes were reduced in preterm-born subjects with and without BPD compared with subjects born at term. Left ventricular mass was comparable (Table). Net PA flow was lower in preterm-born young adults with BPD compared with subjects born at term. For the ventricular curvature ratio, there were no differences between groups, compatible with a lack of right ventricular pressure differences in these groups. Figure 2A shows a lower PA RAC in preterm-born subjects with (62% [56–82]) and without BPD (78% [67–93]) compared with subjects born at term (101% [87–122]), both *P*<0.01. No significant difference was shown between preterm-born subjects with and without BPD (*P*=0.11). Figure 2B illustrates that PA PWV was higher in preterm-born subjects with BPD (2.07 m/s [1.45–3.05]) compared with those without BPD (1.61 m/s [1.18–1.85]; *P*=0.04) and to subjects born at term (1.35 m/s [1.08–2.23]; *P*=0.04). There was no significant difference between preterm-born subjects without BPD and subjects born at term (*P*=0.46). RAC was significantly correlated to maximum workload (r=0.40, *P*=0.002) and peak VO_2_/kg (r=0.43, *P*<0.001), where no significant correlation was found between PWV and peak VO_2_/kg (r=0.38, *P*=0.78) or maximum workload (r=−0.26, *P*=0.85).

**Table.**
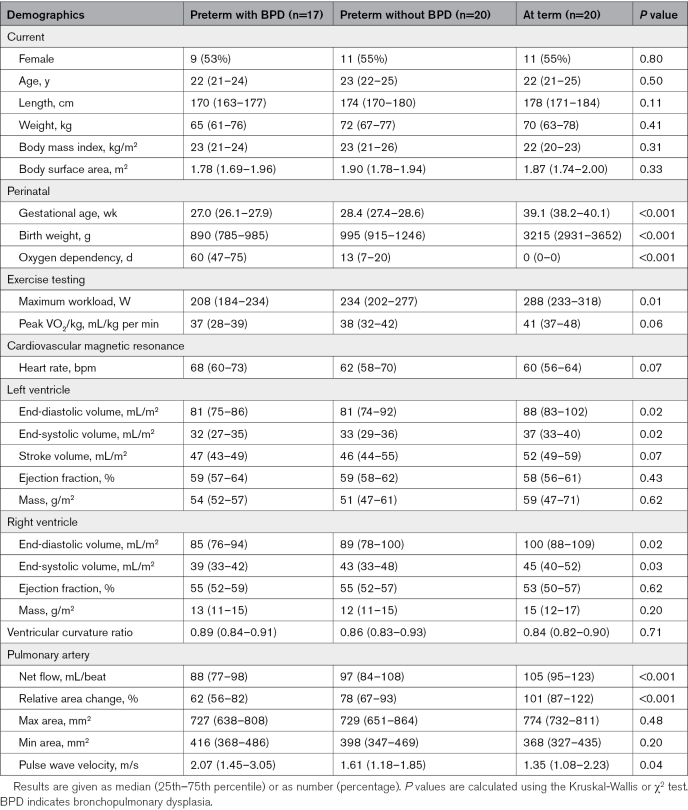
Characteristics and Baseline Measurements of the Study Population

## Discussion

The current study investigated the use of a previously published method^5^ of measuring PWV using CMR to detect proximal PA stiffness in preterm-born young adults with or without BPD, compared with subjects born at term. Preterm-born young adults with BPD showed increased PWV compared with preterm-born young adults without BPD and at-term-born controls. RAC of the PA was decreased in both preterm-born young adults with and without BPD, compared with the at-term-born controls. RAC correlated with parameters of exercise capacity.

BPD is a multifactorial disease characterized by inflammation, endothelial dysfunction, and remodeling of the pulmonary (micro)vasculature.^14^ After intensive care, infants with BPD increasingly reach adulthood.^15^ As stated in the introduction, preterm-born young adults have been shown to have a 5-fold risk of PAH, a detrimental disease despite treatments. Several studies report above-normal resting pulmonary arterial pressures in preterm-born young adults, especially in those with BPD.^16–20^ Secondary hits to the endothelium—such as smoking, aging, and chronic kidney disease—could induce overt PAH later in life.^21^ Early detection and timely treatment of pulmonary vascular disease before overt PAH develops may improve outcomes.^22–24^ Therefore, regular and accurate monitoring of the pulmonary circulation—preferably noninvasively—is crucial to detect early changes in pulmonary vascular disease.^25^

PWV of the PA is a measurement of proximal PA stiffness. Increased systemic PWV is a known predictor of worse prognosis in various sorts of diseases such as heart failure, chronic pulmonary obstructive lung disease, patients receiving extracorporeal membrane oxygenation, and patients with arterial hypertension.^26–29^ Generally, PWV is measured in the aorta and its branches using echocardiography.^30^ However, this is not feasible in the PA because of the anatomy. Therefore, PWV of the PA is often measured using CMR.^31^ Patients with PAH with above-median PWV values have a 3.5-fold risk of mortality compared with those under the median.^10^ When comparing the results of this study to a cohort of Schäfer et al^10^ in patients with PAH and controls, preterm-born subjects with BPD have a PWV lower than PAH patients (2.1 versus 3.2 m/s), while the control values are comparable (1.5 versus 1.6 m/s). Comparing to the largest study analyzing PA stiffness by RAC of Sanz et al is difficult as PA stiffness is very dependent on age.^32,33^ Compared with the control group, patients with pulmonary hypertension had 30% of the RAC, whereas our preterm-born young adults with BPD showed 70% of the RAC compared with age-matched controls.^33^ These findings are highly relevant, as increased proximal PA stiffness may point towards the early stages of developing PAH. Furthermore, increased proximal PA stiffness results in more pulsatile flow in the pulmonary microcirculation, inducing microvascular inflammation, remodeling, and increased pulmonary vascular resistance. In turn, increased pulmonary vascular resistance and PA pressure induce more proximal PA stiffness, entering a vicious cycle.^25^ In a study by Goss et al,^5^ a similar pattern was noted using invasive pressure measurement: preterm-born young adults with BPD showed higher than normal pulmonary arterial pressures, not yet fulfilling the criteria for PAH.^10^ PA stiffness measured by PWV and RAC could be a valuable, noninvasive tool to detect early pulmonary vascular disease in this population.

The subjects of this study were exposed to exercise CMR, as published earlier.^9^ Preterm-born young adults with BPD showed cardiac dysfunction during exercise, as evidenced by a decrease in left ventricular end-diastolic volume, which was not apparent with imaging at rest. This could be explained by left ventricular diastolic dysfunction, right ventricular dysfunction, or increased pulmonary vascular pressures during exercise, which have all been described in premature-born young adult populations.^5,17,34^ Further studies should examine the relationship between PA stiffness and cardiac dysfunction during exercise in preterm-born young adults with BPD.

### Strengths and Limitations

This study assessed a noninvasive method by CMR to analyze proximal PA stiffness in a population at risk for early pulmonary vascular disease but without overt PAH. This method is relatively easy to perform with minimal discomfort for participants. The results indicate that this method is valid to use in a large setting to monitor preterm-born young adults to differentiate patients at risk for PAH. Furthermore, our study control (at-term-born) subjects showed similar PWV values compared with control values in the study by Schäfer et al^10^, thereby indicating the reproducibility of this method. However, before clinical implementation is possible, more research is needed, especially in larger cohorts with longer follow-up. This will serve the purpose of investigating how well these markers can differentiate patients at risk and if changes can be detected with these methods. Preferably, the PWV measurements should be validated with invasive pressure measurements.

It is important to note that the development of BPD postnatally is a gradual process. In this study, preterm-born young adults without BPD did not show all characteristics of increased stiffness, as PWV was not significantly increased, while RAC was decreased. As subject numbers were relatively low, this study is prone to a type II error. A large, population-based study with serial follow-up should confirm if the current study was underpowered to detect differences between preterm-born young adults without BPD and young adults born at term.

### Conclusions

Preterm-born young adults with BPD have increased PA stiffness compared with preterm-born young adults without BPD and at-term-born young adults. Noninvasive measurements of PWV and RAC using CMR to detect PA stiffness could be a valuable method to identify preterm-born young adults at risk for the development of PAH.

## ARTICLE INFORMATION

### Sources of Funding

This study is funded by the Thoraxfoundation project number 22109, Erasmus MC Sophia foundation S12-13, Erasmus MC, Rotterdam, The Netherlands.

### Disclosures

None.

## References

[1] Blencowe H Cousens S Oestergaard MZ Chou D Moller AB Narwal R Adler A Vera Garcia C Rohde S Say L. National, regional, and worldwide estimates of preterm birth rates in the year 2010 with time trends since 1990 for selected countries: a systematic analysis and implications. Lancet. 2012;379:2162–2172. doi: 10.1016/S0140-6736(12)60820-4 22682464 10.1016/S0140-6736(12)60820-4

[2] Jobe AH Bancalari E. Bronchopulmonary dysplasia. Am J Respir Crit Care Med. 2001;163:1723–1729. doi: 10.1164/ajrccm.163.7.2011060 11401896 10.1164/ajrccm.163.7.2011060

[3] Baker CD Abman SH. Impaired pulmonary vascular development in bronchopulmonary dysplasia. Neonatology. 2015;107:344–351. doi: 10.1159/000381129 26044102 10.1159/000381129 PMC4469359

[4] Zysman-Colman Z Tremblay GM Bandeali S Landry JS. Bronchopulmonary dysplasia - trends over three decades. Paediatr Child Health. 2013;18:86–90. doi: 10.1093/pch/18.2.86 24421662 10.1093/pch/18.2.86 PMC3567902

[5] Goss KN Beshish AG Barton GP Haraldsdottir K Levin TS Tetri LH Battiola TJ Mulchrone AM Pegelow DF Palta M. Early pulmonary vascular disease in young adults born preterm. Am J Respir Crit Care Med. 2018;198:1549–1558. doi: 10.1164/rccm.201710-2016OC 29944842 10.1164/rccm.201710-2016OC PMC6298636

[6] Naumburg E Soderstrom L Huber D Axelsson I. Risk factors for pulmonary arterial hypertension in children and young adults. Pediatr Pulmonol. 2017;52:636–641. doi: 10.1002/ppul.23633 27801982 10.1002/ppul.23633

[7] Crump C Howell EA Stroustrup A McLaughlin MA Sundquist J Sundquist K. Association of preterm birth with risk of ischemic heart disease in adulthood. JAMA Pediatr. 2019;173:736–743. doi: 10.1001/jamapediatrics.2019.1327 31157896 10.1001/jamapediatrics.2019.1327 PMC6547251

[8] Agoston-Coldea L Lupu S Mocan T. Pulmonary artery stiffness by cardiac magnetic resonance imaging predicts major adverse cardiovascular events in patients with chronic obstructive pulmonary disease. Sci Rep. 2018;8:14447. doi: 10.1038/s41598-018-32784-6 30262820 10.1038/s41598-018-32784-6 PMC6160404

[9] Steenhorst JJ Helbing WA van Genuchten WJ Bowen DJ van den Bosch A van der Velde N Kamphuis LS Merkus D Reiss IKM Hirsch A. Cardiac dysfunction during exercise in young adults with bronchopulmonary dysplasia. ERJ Open Res. 2024;10:00501–02023. doi: 10.1183/23120541.00501-2023 38887679 10.1183/23120541.00501-2023 PMC11181055

[10] Schäfer M Wilson N Ivy DD Ing R Abman S Browne LP Morgan G Ross M McLennan D Barker AJ. Noninvasive wave intensity analysis predicts functional worsening in children with pulmonary arterial hypertension. Am J Physiol Heart Circ Physiol. 2018;315:H968–H977. doi: 10.1152/ajpheart.00227.2018 30004811 10.1152/ajpheart.00227.2018 PMC6737454

[11] R Core Team R. R: A Language and Environment for Statistical Computing. 2013.

[12] Zeileis A Grothendieck G Ryan JA Andrews F Zeileis MA. Package “zoo.” R Package Version. 2014:1.7-12.

[13] Critser PJ Higano NS Lang SM Kingma PS Fleck RJ Hirsch R Taylor MD Woods JC. Cardiovascular magnetic resonance imaging derived septal curvature in neonates with bronchopulmonary dysplasia associated pulmonary hypertension. J Cardiovasc Magn Reson. 2020;22:50. doi: 10.1186/s12968-020-00643-x 32698897 10.1186/s12968-020-00643-x PMC7376717

[14] Collins JJP Tibboel D de Kleer IM Reiss IKM Rottier RJ. The future of bronchopulmonary dysplasia: emerging pathophysiological concepts and potential new avenues of treatment. Front Med (Lausanne). 2017;4:61. doi: 10.3389/fmed.2017.00061 28589122 10.3389/fmed.2017.00061 PMC5439211

[15] Arjaans S Haarman MG Roofthooft MTR Fries MWF Kooi EMW Bos AF Berger RMF. Fate of pulmonary hypertension associated with bronchopulmonary dysplasia beyond 36 weeks postmenstrual age. Arch Dis Child Fetal Neonatal Ed. 2021;106:45–50. doi: 10.1136/archdischild-2019-318531 32571832 10.1136/archdischild-2019-318531 PMC7788204

[16] Lewandowski AJ. The preterm (right) heart: does bronchopulmonary dysplasia play a unique role in long-term remodeling? Chest. 2021;160:27–28. doi: 10.1016/j.chest.2021.02.039 34246369 10.1016/j.chest.2021.02.039

[17] Lewandowski AJ Bradlow WM Augustine D Davis EF Francis J Singhal A Lucas A Neubauer S McCormick K Leeson P. Right ventricular systolic dysfunction in young adults born preterm. Circulation. 2013;128:713–720. doi: 10.1161/CIRCULATIONAHA.113.002583 23940387 10.1161/CIRCULATIONAHA.113.002583

[18] Barton GP Corrado PA Francois CJ Chesler NC Eldridge MW Wieben O Goss KN. Exaggerated cardiac contractile response to hypoxia in adults born preterm. J Clin Med. 2021;10:1166. doi: 10.3390/jcm10061166 33802149 10.3390/jcm10061166 PMC7999333

[19] Corrado PA Barton GP Macdonald JA Francois CJ Eldridge MW Goss KN Wieben O. Altered right ventricular filling at four-dimensional flow MRI in young adults born prematurely. Radiol Cardiothorac Imaging. 2021;3:e200618. doi: 10.1148/ryct.2021200618 34250493 10.1148/ryct.2021200618 PMC8259661

[20] Macdonald JA Roberts GS Corrado PA Beshish AG Haraldsdottir K Barton GP Goss KN Eldridge MW Francois CJ Wieben O. Exercise-induced irregular right heart flow dynamics in adolescents and young adults born preterm. J Cardiovasc Magn Reson. 2021;23:116. doi: 10.1186/s12968-021-00816-2 34670573 10.1186/s12968-021-00816-2 PMC8529801

[21] Goss K. Long-term pulmonary vascular consequences of perinatal insults. J Physiol. 2019;597:1175–1184. doi: 10.1113/JP275859 30067297 10.1113/JP275859 PMC6375870

[22] Berger RM Beghetti M Humpl T Raskob GE Ivy DD Jing ZC Bonnet D Schulze-Neick I Barst RJ. Clinical features of paediatric pulmonary hypertension: a registry study. Lancet. 2012;379:537–546. doi: 10.1016/S0140-6736(11)61621-8 22240409 10.1016/S0140-6736(11)61621-8 PMC3426911

[23] Galie N Rubin L Hoeper M Jansa P Al-Hiti H Meyer G Chiossi E Kusic-Pajic A Simonneau G. Treatment of patients with mildly symptomatic pulmonary arterial hypertension with bosentan (EARLY study): a double-blind, randomised controlled trial. Lancet. 2008;371:2093–2100. doi: 10.1016/S0140-6736(08)60919-8 18572079 10.1016/S0140-6736(08)60919-8

[24] Lau EM Manes A Celermajer DS Galie N. Early detection of pulmonary vascular disease in pulmonary arterial hypertension: time to move forward. Eur Heart J. 2011;32:2489–2498. doi: 10.1093/eurheartj/ehr160 21616950 10.1093/eurheartj/ehr160

[25] Sun W Chan SY. Pulmonary arterial stiffness: an early and pervasive driver of pulmonary arterial hypertension. Front Med (Lausanne). 2018;5:204. doi: 10.3389/fmed.2018.00204 30073166 10.3389/fmed.2018.00204 PMC6058030

[26] Malik MI Nagpal D. Estimated pulse-wave velocity predicts survival in patients requiring extracorporeal membrane oxygenation. Perfusion. 2024;39:344–352. doi: 10.1177/02676591221141963 36419384 10.1177/02676591221141963

[27] Bonapace S Rossi A Cicoira M Targher G Valbusa F Benetos A Vassanelli C. Increased aortic pulse wave velocity as measured by echocardiography is strongly associated with poor prognosis in patients with heart failure. J Am Soc Echocardiogr. 2013;26:714–720. doi: 10.1016/j.echo.2013.03.022 23676208 10.1016/j.echo.2013.03.022

[28] Valencia-Hernandez CA Lindbohm JV Shipley MJ Wilkinson IB McEniery CM Ahmadi-Abhari S Singh-Manoux A Kivimaki M Brunner EJ. Aortic pulse wave velocity as adjunct risk marker for assessing cardiovascular disease risk: prospective study. Hypertension. 2022;79:836–843. doi: 10.1161/HYPERTENSIONAHA.121.17589 35139665 10.1161/HYPERTENSIONAHA.121.17589 PMC9148390

[29] Coutinho T Turner ST Kullo IJ. Aortic pulse wave velocity is associated with measures of subclinical target organ damage. JACC Cardiovasc Imaging. 2011;4:754–761. doi: 10.1016/j.jcmg.2011.04.011 21757166 10.1016/j.jcmg.2011.04.011 PMC3862768

[30] Asmar R Benetos A Topouchian J Laurent P Pannier B Brisac AM Target R Levy BI. Assessment of arterial distensibility by automatic pulse wave velocity measurement. Validation and clinical application studies. Hypertension. 1995;26:485–490. doi: 10.1161/01.hyp.26.3.485 7649586 10.1161/01.hyp.26.3.485

[31] Quail MA Knight DS Steeden JA Taelman L Moledina S Taylor AM Segers P Coghlan GJ Muthurangu V. Noninvasive pulmonary artery wave intensity analysis in pulmonary hypertension. Am J Physiol Heart Circ Physiol. 2015;308:H1603–H1611. doi: 10.1152/ajpheart.00480.2014 25659483 10.1152/ajpheart.00480.2014 PMC4469876

[32] Horvat D Zlibut A Orzan RI Cionca C Muresan ID Mocan T Revnic R Agoston-Coldea L. Aging influences pulmonary artery flow and stiffness in healthy individuals: non-invasive assessment using cardiac MRI. Clin Radiol. 2021;76:161.e19–161.e28. doi: 10.1016/j.crad.2020.09.021 10.1016/j.crad.2020.09.021 33109351

[33] Sanz J Kariisa M Dellegrottaglie S Prat-Gonzalez S Garcia MJ Fuster V Rajagopalan S. Evaluation of pulmonary artery stiffness in pulmonary hypertension with cardiac magnetic resonance. JACC Cardiovasc Imaging. 2009;2:286–295. doi: 10.1016/j.jcmg.2008.08.007 19356573 10.1016/j.jcmg.2008.08.007

[34] Lewandowski AJ Raman B Bertagnolli M Mohamed A Williamson W Pelado JL McCance A Lapidaire W Neubauer S Leeson P. Association of preterm birth with myocardial fibrosis and diastolic dysfunction in young adulthood. J Am Coll Cardiol. 2021;78:683–692. doi: 10.1016/j.jacc.2021.05.053 34384550 10.1016/j.jacc.2021.05.053 PMC8363934

